# Human papillomavirus vaccination status among the urban and rural married women of Bangladesh: An analytical cross-sectional study

**DOI:** 10.1016/j.pmedr.2026.103464

**Published:** 2026-04-03

**Authors:** Md Tamzidus Sifat, Maliha Malbika Mimu, Abul Masud Md. Nurul Karim, Shafia Shaheen, Obaidullah Ibn Raquib

**Affiliations:** a Department of Epidemiology, National Institute of Preventive and Social Medicine (NIPSOM), Mohakhali, Dhaka, 1212, Bangladesh; b Shaheed Suhrawardy Medical College Hospital, Sher-e-Bangla Nagar, Dhaka, 1207, Bangladesh; c Department of Biostatistics, National Institute of Preventive and Social Medicine (NIPSOM), Mohakhali, Dhaka, 1212, Bangladesh

**Keywords:** Human papillomavirus, Vaccination, Cervical cancer, Women's health, Health disparities, Geographic disparities, Female reproductive health

## Abstract

**Objective:**

Among Bangladeshi women, cervical cancer ranks in the second place of the most common cancers whereas it's ranked fourth worldwide. Vaccination against Human Papillomavirus (HPV) is the most effective way of reducing the infection and incidence of cervical cancer. The study strived to assess the HPV vaccination status among the married women of Bangladesh and identify possible barriers to vaccination.

**Methods:**

Analytical cross-sectional study performed between January 1st to December 31st 2023. Data were collected through face-to-face interviews using a semi-structured questionnaire. Study population were conveniently selected by domiciliary visit from selected urban and rural areas of Dhaka and Jamalpur districts. Married women over 18 years of age were selected conveniently, who provided informed written consent.

**Results:**

Out of the 384 respondents, only 36 took the HPV vaccine and location breakdown showed 25 urban and 11 rural women received the vaccine. *Chi-squared* test showed statistically significant difference, χ2(1, *N* = 384) = 6.01, *p* < .02, meaning urban women are more likely to get vaccinated. Principal barriers found were “Lack of knowledge” (33.05%), “Lack of Interest” (21.55%) and “Financial Barrier” (20.98%).

**Conclusions:**

Health education, international collaboration to boost the vaccination coverage and subsidizing the vaccines are required to achieve the WHO's cervical cancer eradication goal.

## Highlights

• Cervical cancer is the second most prevalent cancer among Bangladeshi women.
• Urban married women are more likely to get vaccinated than rural counterparts.
• Lack of knowledge, interest and financial constraint are principal barriers.
• Urban women are more likely to pursue higher education, and are more educated.
• Women achieving higher levels of education are more likely to get vaccinated.

## 1 Introduction

Cervical cancer is the fourth most common cancer in terms of incidence and mortality worldwide with incidence rate of 6.8%, among all cancers (Bray et al., 2024). Cervical cancer is defined as the cancer that starts at the cells lining the cervix. The cervix is the lower; narrow end of the uterus; connecting the uterus to the vagina (What Is Cervical Cancer?, 2022). Among all the cases and deaths in 2022; about 94% occurred in low and middle-income countries. The age-standardized incidence rate is 19.1 per 1;00;000 among south Asian women; compared to 12.1 per 1;00;000 women of the developed world; and the age-standardized mortality is 12.4 vs 4.8 per 1;00;000 women when comparing those same demographic groups (Bray et al., 2024). The situation in Bangladesh is worse; with cervical cancer being the second most common cancer with the proportion around 12% of all cancers (Uddin et al., 2023).

Human Papillomavirus (HPV) is a necessary, but not sufficient, cause of cervical cancer (Walboomers et al., 1999). Of the known 448 HPV genotypes; 12 are categorically classified by the “*IARC monographs*” as “Group 1” carcinogen (IARC Working Group on the Evaluation of Carcinogenic Risks to Humans, 2007). HPV is a small, non-enveloped DNA virus that infects skin or mucosal cells. At least 13 genotypes can cause cervical cancer and are associated with other anogenital, head and neck cancers. The “HPV 16” and “HPV 18” are high-risk genotypes, causing about 70% of the cervical cancers worldwide (Human Papillomavirus (HPV), 2023a). Risk factors of progression to cancer include the oncogenicity of the HPV genotype, immunity, presence of other sexually transmitted infections, parity, early first pregnancy, hormonal contraceptives and smoking (Cervical cancer, 2025). Vaccination against Human Papillomavirus, screening for suspicious lesions and treatment of suspected pre-cancerous lesions are the most cost-effective ways to prevent cervical cancer (Cervical cancer, 2025).

At the 73rd World Health Assembly, the WHO adopted the cervical cancer elimination initiative, having national targets dubbed with the acronym “*90‐70-90 target*”, for countries to be on the path of eliminating cervical cancer by the year 2030. The pillars of the target are: 90% HPV vaccination coverage for eligible girls, 70% screening coverage with a high-performance test and 90% of women with a positive screening test or a cervical lesion managed appropriately. Prevention of cervical cancer is integral to reaching the Sustainable Development Goal (SDG), both, health (SDG3) and gender equality (SDG5) (WHO, 2021). As of 2025, eight vaccines are licensed and five available worldwide for Human Papillomavirus (Cervical cancer, 2025). USFDA approved vaccines are bivalent “*Cervarix™*”, tetravalent “*Gardasil™*” and nine-valent “*Gardasil9™*” (Use of 9-Valent Human Papillomavirus (HPV), 2015). Of these, the former two are available in Bangladesh but have become exceedingly rare (Reuters, 2018). The government-initiated vaccination campaign for the “under-15” girls specified in the global strategy for elimination of cervical cancer (Human Papillomavirus (HPV), 2023b). This study tried to explore the possible barriers to vaccination among urban and rural married women and also get a picture of the vaccination status of this population so we could predict the magnitude of intervention required for cancer prevention.

## 2 Methods

This analytical cross-sectional study was reviewed and cleared for commencing by the Institutional Review Board of the National Institute of Preventive and Social Medicine (NIPSOM), Dhaka, Bangladesh. Respondents were informed about the study's purpose, including preservation of anonymity and voluntary nature of their response. Participants provided informed consent. It followed Strengthening the Reporting of Observational Studies in Epidemiology (STROBE) guidelines for cross-sectional studies.

### 2.1 Study design and population

This study was conducted to try and find out the Human Papillomavirus vaccination status among the urban and rural married women of Bangladesh. It was conducted in selected urban and rural areas of Dhaka and Jamalpur district, chosen for their convenience. All the urban and rural married women of Bangladesh were the possible population pool, who satisfied the inclusion criterion; being married. Sample size was calculated using the following formula,

$$n=\frac{z^{2}[\{P_{1}\left(1-P_{1)}\right\}+\left\{P_{2}\left(1-P_{2}\right)\right\}]}{d^{2.}}$$

Here, n, being the number of respondents in each group. The calculated sample size in each group was 192 and total sample size, 384. The sampling technique for selection of study places was convenient. Respondent selection was also convenient, going door-to-door to the homes and workplaces for data collection, until 192 urban and 192 rural respondents were achieved. The target, 192 urban women were reached, approaching 217 women and 192 rural women, approaching 209 women who consented for the interview. Data was collected by a pre-tested semi-structured questionnaire which was prepared in both, Bengali and English. The Bengali questionnaire along with a Bengali informed written consent were used for data collection. Data was collected by face-to-face interview ensuring privacy and confidentiality.

### 2.2 Measures

After collection, data was checked for consistency and completeness, then cleaned, edited and entered in software. Data was re-coded, managing missing values through frequency run. The analysis plan was developed according to the objective of the study. New variables were computed as per requirement of analysis. Some variables were categorized which were primarily measured in ratio scale.

### 2.3 Statistical analysis

Demographic, socio-economic and associated factors related characteristics were shown using frequency tables. Descriptive statistics were calculated separately for urban and rural women and compared. Inferential analysis was done according to the objective of the study. The proportion of urban and rural married women who took the vaccine was calculated, the women were selected as the independent variable and vaccination status as the dependent variable. Between these, a *chi-squared* test of independence with α = 0.05 was used to assess whether residency distribution and vaccination of urban and rural married women were significantly associated. A *P-value of* < 0.05 was considered statistically significant. Zero cells had expected count of less than five, so no “Yates correction” was needed. The minimum expected count was 18. Phi (φ) coefficient was used to assess the effect size due to both data being dichotomous. *Binary logistic regression* was done following the chi-squared test. Data were analyzed using *SPSS STATISTICS (Version 27)*.

## 3 Results

The cross-sectional study was performed to try to find out the HPV vaccination status among the urban and rural married women of Bangladesh, in which 384 women divided evenly from urban and rural areas were taken as representative respondents. The mean age (SD) of the respondents (*n* = 384) was 34.03 (± 10.66) years, with the median value, 32 years. Distribution according to religious beliefs revealed 361 Muslims, 19 Hindus, three Christians and only one Buddhist.

Marital status data showed, among the urban women, 176 were married, five divorced and 11 widowed. Among the rural women, 183 were married, four divorced and five were widowed. Educational qualification of the women showed 31 urban vs 69 rural women whose level were up to primary school completed (Class five completed for Bangladesh), 51 urban vs 96 rural women whose level were above primary up to higher secondary school completed (Class 12 completed) and 110 urban vs 27 rural women whose level were above higher secondary up to post-graduation completed, showing urban women are more likely to pursue higher education. Also, 149 urban vs 119 rural women lived in a nuclear family whereas 43 urban vs 73 rural women lived in an extended family. Occupation status of the respondents is presented in Table 1.

**Table 1:** Descriptive socio-demographic characteristics among the urban (*n* = 192) and rural (n = 192) married women of “Dhaka” district and “Jamalpur” district of Bangladesh, January – December 2023.

Measure		Urban women F (%)	Rural women F (%)
Educational Qualification of the Respondents	Up to primary school completed	31 (16.15)	69 (35.93)
	Above primary up to higher secondary school completed	51 (26.56)	96 (50)
	Above higher secondary up to post-graduation completed	110 (57.29)	27 (14.06)

Family Type of the Respondents	Nuclear family	149 (77.60)	119 (61.98)
	Extended family	43 (22.40)	73 (38.02)

Family Living Arrangement of the Respondents	Living with husband	165 (85.94)	154 (80.21)
	Living away from husband	27 (14.06)	38 (19.79)

Field of Occupation of the Respondents	Housewife	93 (48.44)	94 (48.96)
	Service	70 (36.46)	25 (13.02)
	Laborer	14 (7.29)	26 (13.54)
	Business	7 (3.65)	19 (9.90)
	Others*	8 (4.16)	28 (14.58)

Estimated Monthly Family Income of the Respondents (Amount shown in Bangladeshi Taka)	1 to 20,000	17 (8.85)	38 (19.79)
	20,001 to 40,000	62 (32.29)	127 (66.15)
	40,001 to 60,000	47 (24.48)	20 (10.42)
	60,001 to 80,000	53 (27.60)	5 (2.60)
	80,001 to 100,000	10 (5.22)	2 (1.04)
	100,001 to 120,000	1 (0.52)	0 (0)
	Above 120,000	2 (1.04)	0 (0)

Note: n: number of urban and rural married women in the study, F: frequency of each response, %: percentage, ^⁎^ The category branded as “Others” exclusively incorporated students of various education levels actively not pursuing any other jobs, they incorporated students of secondary, higher secondary, graduate and post-graduate students, Empty cells are not because of missing data, but due to the design of the table.

Vaccination status with respect to occupation of the respondents show that a mere five of the 187 housewives had taken the HPV vaccine. The maximum vaccinated came from the “service” category, which included both government and non-government service holders and 21 of the 95 respondents responded positively to taking the vaccine. The most concerning statistics came from the “Laborer” category. None of the 40 respondents had taken the vaccine. Lastly, three of the various 23 businesspersons and seven of the 29 respondents categorized as “Others” who were almost exclusively students, got the vaccine.

Table 2 illustrates the knowledge regarding cervical cancer and healthcare seeking behavior patterns among the respondents. The majority of the urban women didn't go for regular health check-up and didn't have any clinical or laboratory examination for the screening of cervical cancer, which is one of the targets of the WHO for eradication. Although, urban women are ahead of rural women by a huge margin in all these criteria.

**Table 2:** Descriptive distribution with comparison of the healthcare seeking behavior pattern and cervical cancer knowledge status among the urban (*n* = 192) and rural (n = 192) married women of “Dhaka” district and “Jamalpur” district of Bangladesh, January – December 2023.

Measure		Urban women F (%)	Rural women F (%)
Knowledge of Cervical Cancer among the Respondents	Knows about cervical cancer	180 (93.75)	143 (74.48)
	Doesn't know about cervical cancer	12 (6.25)	49 (25.52)

Regular Medical Check-up Status the Respondents	Goes for regular medical check-up	85 (44.27)	25 (13.02)
	Doesn't go for regular medical check-up	107 (55.73)	167 (86.98)

Clinical Examination Status for Cervical Cancer among the Respondents	Previously had clinical examination	32 (16.67)	9 (4.69)
	Previously didn't have clinical examination	160 (83.33)	183 (95.31)

Laboratory Test Status for Cervical Cancer among the Respondents	Previously had laboratory test	12 (6.25)	3 (1.56)
	Previously didn't have laboratory test	180 (93.75)	189 (98.44)

Knowledge of Human Papillomavirus Causing Cervical Cancer among the Respondents*	Knows about Human Papillomavirus causing cervical cancer	140 (72.92)	81 (42.19)
	Doesn't know about Human Papillomavirus cervical causing cancer	52 (27.08)	111 (57.81)

Knowledge of Human Papillomavirus Vaccine Availability among the Respondents	Knows about HPV vaccine availability	156 (81.25)	116 (60.42)
	Doesn't know about HPV vaccine availability	36 (18.75)	76 (39.58)

Human Papillomavirus Vaccination Status among the Respondents	Taken the Human Papilloma Virus vaccine	25 (13.02)	11 (5.73)
	Didn't take the Human Papilloma Virus vaccine	167 (86.98)	181 (94.27)

Note: n: number of urban and rural married women in the study, F: frequency of each response, %: percentage, * Knowledge about the Human Papillomavirus as a microorganism among the respondents is described further in the “Results” section, Empty cells are not because of missing data, but due to the design of the table.

Furthermore, the table demonstrates the difference in raw numbers between the urban and rural women regarding knowledge about cervical cancer, knowledge about HPV causing cervical cancer, vaccine for this virus and the status of vaccination. It's important to mention that 221 respondents knew that “a microorganism” can cause cervical cancer; nearly half of them couldn't specifically name the microorganism (i.e. HPV), but they are considered knowledgeable about HPV in this study.

The number of respondents who answered positively about knowing cervical cancer were 323. Among them, the highest proportion (37.77%) learned from a healthcare worker, both government and private healthcare institutions included. “Relatives” occupy the second position (19.50%) as the knowledge source. Conventional media like TV, Radio and Newspapers were the source for 9.58% respondents and social media provided 11.76% respondents with the information. The “Friends”, composed of various acquaintances, was the third common response (13.31%). Social workers like different non-government organizations (NGO) personnel provided information to about 8.05% respondents.

Table 3 provides the statistical analyses of knowledge and vaccination status of the urban and rural women with regards to their location of residence and level of education, using the *chi-squared* test. The study finds; urban women are more likely to know about cervical cancer than their rural counterparts, χ2 (1, *N* = 384) = 26.68, *p* < .00. Also, urban women are more likely to know about a microorganism (i.e. HPV) causing cervical cancer χ2 (1, *N* = 384) = 37.11, *p* < .00 and furthermore, know about the fact that, there are vaccines available against the organism causing cervical cancer χ2 (1, *N* = 384) = 20.17, *p* < .00.

**Table 3:** Descriptive distribution and comparison of the cervical cancer vaccination status among the urban (*n* = 192) and rural (n = 192) married women of “Dhaka” district and “Jamalpur” district of Bangladesh, January – December 2023.

Measure			Results			
			df	Test of Significance (χ^2^)	*P* value	Effect size
Residence Distribution by Location	Knowledge of Cervical Cancer among the respondents					
	Yes	No				
Urban	180	12	1	26.68	<0.00	φ = 0.30*
Rural	143	49				

Residence Distribution by Location	Knowledge of Human Papillomavirus Causing Cervical Cancer among the respondents					
	Yes	No				
Urban	140	52	1	37.11	<0.00	φ = 0.31*
Rural	81	111				

Residence Distribution by Location	Knowledge of Human Papillomavirus Vaccine Availability among the respondents					
	Yes	No				
Urban	156	36	1	20.17	<0.00	φ = 0.23*
Rural	116	76				

Residence Distribution by Location	Human Papillomavirus Vaccination Status among the respondents					
	Yes	No				
Urban	25	167	1	6.01	<0.02	φ = 0.13*
Rural	11	181				

Level of Education	Knowledge of Cervical Cancer among the respondents					
	Yes	No				
Up to primary level completed	57	43	2	82.35	<0.00	V = 0.46**
Above primary up to higher secondary level completed	129	18				
Above higher secondary up to post-graduate level completed	137	0				

Level of Education	Human Papillomavirus Vaccination Status among the respondents					
	Yes	No				
Up to primary level completed	1	99	2	19.57	<0.00	V = 0.23**
Above primary up to higher secondary level completed	11	136				
Above higher secondary up to post-graduate level completed	24	113				

Note: P-value is obtained from the *chi-square* (χ^2^) test, df is degree of freedom, ^⁎^ Phi (φ) coefficient shows the strength of association when degree of freedom (df) is 1, ^⁎⁎^ Cramer's V shows the strength of association when degree of freedom (df) is 2, Empty cells are not because of missing data, but due to the design of the table.

The primary objective of the study was to find out if the vaccination status significantly differed between the urban and rural married women, and the data showed that urban women are more likely to be vaccinated, χ2 (1, *N* = 384) = 6.01, *p* < .02. When level of education was compared with the respondent's knowledge of cervical cancer and vaccination status, they were found to be statistically significantly different; showing, as the level of education increased, so did the knowledge of cervical cancer, χ2 (2, *N* = 384) = 82.35, *p* < .00 and their vaccination status, χ2 (2, *N* = 384) = 19.57, *p* < .00 respectively. Regression analysis showed slight increase in vaccination with increasing age (OR = 1.03) and urban married women are likely to get vaccinated more than the rural married women (OR = 1.56), although the results are not statistically significant. Table 5

Table 4 illustrates the locations from where the individuals procured the vaccines, and maximum respondents got it from a healthcare facility. “Sandhani”, a voluntary blood-donation organization run by medical students, also provided eight respondents and “nine” women procured vaccines from a pharmacy. Four respondents got the vaccine as part of the school vaccination campaign through the EPI initiative by the government with the aid from the WHO. The table also provide details on why the non-vaccinated respondents were averse from taking the vaccine with principal barriers found to be causes like “Lack of knowledge”, “Lack of Interest” and “Financial Barrier”.

**Table 4:** Descriptive distribution with comparison of attitude towards vaccination and vaccine procurement location among the urban (n = 192) and rural (n = 192) married women of “Dhaka” district and “Jamalpur” district of Bangladesh, January – December 2023.

Measure		Non-vaccinated, F = 348 (%)	Vaccinated, F = 36 (%)
Motivational Support for Taking Vaccine	Healthcare workers		16 (44.44)
	Relatives		11 (30.56)
	Friends		9 (25)

Location of Vaccine Procurement	Healthcare facility		15 (41.67)
	Sandhani		8 (22.22)
	Pharmacy		9 (25)
	School vaccine campaign		4 (11.11)

Main Reason for Not Taking the Vaccine	Lack of knowledge about vaccine	115 (33.05)	
	Lack of interest in taking vaccine	75 (21.55)	
	Financial barrier in purchasing vaccine	73 (20.98)	
	Fear of needle/vaccine	22 (6.32)	
	Distrust of vaccine**	13 (3.74)	
	Lack of vaccine availability near the respondent	22 (6.32)	
	Pregnancy	4 (1.15)	
	Fear of vaccine side effects	12 (3.45)	
	Belief that vaccine isn't needed***	11 (3.16)	
	Other*	1 (0.29)	

Consideration of Vaccination Following Interview	Yes	151 (43.39)	
	No	74 (21.26)	
	Not sure	78 (22.41)	
	Need more information	29 (8.33)	
	No answer	16 (4.60)	

Note: n: number of urban and rural married women in the study, F: frequency of each response, %: percentage, ^⁎^ The respondent in “Other” category had “Hysterectomy” done, Empty cells are not because of missing data, but due to the design of the table, ** Distrust of vaccine means the respondent's distrust of the science behind the vaccine and the distrust for the government and other authorities motive to push for vaccination, *** Belief that vaccine isn't needed means the religious belief of the respondents, who believe that, God or a higher power is sufficient for preventing disease rather than any vaccines.

**Table 5:** Binary logistic regression table of the factors affecting Human Papillomavirus vaccinations status among the among the urban (n = 192) and rural (n = 192) married women of “Dhaka” district and “Jamalpur” district of Bangladesh, January – December 2023.

Measures	B	Sig.	Odds Ratio (OR)	95% CI for OR	
				Lower	Upper
Age of the Respondents	0.03	0.15	1.03	0.98	1.07
Residency Distribution by Location (1)	0.44	0.27	1.56	0.70	3.43
Know that there is vaccine available against Human Papillomavirus (1)	- 0.09	1.00	0.91	0.00	

Note: (1) No was taken as the reference value of the dependent variable, C.I.: Confidence Interval, Sig.: Significance.

## 4 Discussion

This study provides a small, albeit relatively representative population-based data regarding cervical cancer vaccination status of a third-world country. Age is an important factor contributing to the increase in prevalence of malignancies. It takes 15–20 years in women for the abnormal cells to become cancer, but in immunocompromised women, this can occur earlier (Cervical cancer, 2025). Mean age of the respondents was 34.03 years ±10.66 years with the range being 18–61 years. Similar studies done regarding knowledge and acceptance of HPV vaccine found comparable mean age (Bhuiyan et al., 2018; Islam et al., 2018). Another study found that lack of knowledge is a leading cause of growing cervical cancer incidence among similar age groups (Alam et al., 2022). Because the married women with increasing age are in increased risk of having cervical cancer (Cervical cancer, 2025); this age group should be one of the most important target populations for vaccination. Mhaske-et-al. found that the prevalence of cervical dysplasia was 23% among women who was married between 11 and 14 years of age; followed by 16.56% between 15 and 18 years and 9.09% between 19 and 22 years. Also; women whose husbands previously married or had sexual partners; had increased risk of developing cervical cancer (Mhaske et al., 2011). So; vaccination would give these married women extra protection against developing cervical cancer by reducing the chance of contracting HPV from their husbands because men can be carriers of HPV (Schneider et al., 1988). This is supported by findings of similar studies (The PLOS ONE Staff, 2014) which found women who had two or more sexual partners in their lifetime are four times more likely to get HPV infection than women who only had one sexual partner.

Another study (Mhaske et al., 2011) showed that high number of parity and early age of child bearing, too, increases the risk of cervical cancer. Also, (Islam et al., 2018) found that only urban women and not the rural women knew that parity or high count of childbirth is a risk factor of cervical cancer. To make matters worse; in rural women; it was found in another study (The PLOS ONE Staff, 2014) that “parity” and “husbands residency location status” were associated with increased HPV infection for rural women only.

Another study (Islam et al., 2018) identified education as a significant factor for cervical cancer knowledge among urban women. Urban women having primary education had 2.5 times odds and secondary education or above had almost four times increased odds of hearing about cervical cancer when compared to urban women with no education. No significant effect of education was observed among rural women (Islam et al., 2018). A different study found urban women are more likely to know about cervical cancer than rural women; and women with university education are 1.5 times more likely to know than women with lower level or no education (Alam et al., 2022). We also found a significant relationship between education level and knowledge of cervical cancer among urban and rural women; mirroring the study (Alam et al., 2022), showing urban women are more likely to know about cervical cancer than rural women.

The mean monthly family income (SD) was 41,578.13 (±22,281.70) Bangladeshi Taka (BDT) with the median income 35,000.00 BDT, showing similar results to previous studies and statistical data (Bhuiyan et al., 2018; bdnews24.com, 2024). Our study revealed 319 of the 384 respondents living with their husbands. When it came to living away from husband; the 65 respondents also included women who have been widowed. The living arrangement is important for HPV infection as discussed previously; which showed husbands residency status was associated with HPV infection for rural women only (The PLOS ONE Staff, 2014).

A key finding (The PLOS ONE Staff, 2014) was; occupation had the strongest effect on HPV infection for urban women. Urban women who were working as housemaids or garment workers were at higher risk of any HPV infection compared to those who reported being housewives. The risk doubled for infection with high-risk-HPV subtypes for these two groups of women. As our study found that none of the 40 “laborer” women had taken the HPV vaccine; and they are more likely to get HPV infection compared to “housewives” and the probability doubles for the high-risk viral genotypes (The PLOS ONE Staff, 2014), our study recommends that “The Laborers” should be a first-priority target population for HPV vaccination programs. As, most of the students took their vaccine from the school-based vaccine campaign the government initiated with the help of the WHO (Human Papillomavirus (HPV), 2023b), vaccination campaign should be undertaken for the destitute women including the laborers, housemaids and garments workers etc., as the HPV vaccine was found effective against all four vaccine susceptible types in young women vaccinated after age 12. However, vaccinated women had a higher infection prevalence with high-risk nonvaccine genotypes, suggesting that there may be benefit from newer vaccines covering additional genotypes (Guo et al., 2015).

We found slightly above one-quarter of the respondents had regular medical check-ups. Just over 10% of women had their cervix checked clinically. The most concerning statistics was that, only 3.91% of the respondents had any laboratory examination like “Pap-smear”, even though the WHO targets to screen 70% of all eligible women at least twice in their lifetimes with high-performance screening investigations (WHO, 2021). Our result mimics other studies, which found only 6.90% of their respondents had had *Pap-smear* for cervical cancer screening (Bhuiyan et al., 2018). Only 36 of the 384 respondents confirmed taking the vaccine. The vast majority, 348 out of 384, did not take any of the vaccines. Procurement data shows, only four participants got the vaccine from government-initiated school vaccine campaigns, and the rest got the vaccine through out-of-pocket expenditure. Strengthening voluntary organizations like the “Sandhani” might provide women with easier access to vaccination.

Lack of knowledge about vaccines, lack of interest in taking vaccines and financial barriers were the most common answers given when asked about the “main reason” for not getting vaccinated. Lack of availability was the answer of just above 6 % of the respondents. Superstitions like “Distrust of vaccine” (i.e. distrust for authorities and mandates), “Belief that vaccine is not needed” (i.e. religious belief of the respondents), perceived “side effects” of vaccine following hearing misinformation about COVID-19 vaccine and “fear of needles” combined, prevented almost one-sixth respondents from getting vaccinated. To reduce this number, health promotion and health education is required. Healthcare workers, friends and relatives motivated the respondents in taking the vaccine. The only group absent in this answer was “social workers”. Stakeholders should initiate dialogs with NGO's, whose social workers perform community-based healthy practice teaching, stimulating their workers to motivate more people.

## 5 Conclusion

Our findings suggest; urban women are more likely to take the HPV vaccine compared to their rural counterparts. School vaccination campaigns targeting 10–14-year-old girls are not enough because the married women comprise a significant proportion of the population, who are the current and future mothers, needed to motivate the next generation of women to take the vaccine. The major barriers to vaccination are lack of knowledge about vaccines, superstitions against vaccines and high cost of the vaccine in an economically strained country. The government should try subsidizing the vaccines so that people don't suffer the economic burden, and also should carry out health promotion and health education programs, educating the population about the benefits of vaccines so that the lack of knowledge and superstitions can be eradicated. Urban women are more likely to pursue higher education, and comparatively more educated, so emphasis is needed in rural areas to promote women's education. Women with higher education levels are more likely to get vaccinated, so promoting education will be important in increasing vaccination coverage. Government and private partnership are needed in reaching these goals and ultimately the WHO's goal of eradicating cervical cancer.

## CRediT authorship contribution statement

**Md Tamzidus Sifat:** Writing – review & editing, Writing – original draft, Visualization, Resources, Methodology, Investigation, Formal analysis, Data curation, Conceptualization. **Maliha Malbika Mimu:** Writing – review & editing, Writing – original draft, Resources, Investigation, Data curation, Conceptualization, Methodology. **Abul Masud Md. Nurul Karim:** Writing – review & editing, Supervision, Project administration, Methodology, Conceptualization, Data curation. **Shafia Shaheen:** Writing – review & editing, Supervision, Methodology, Formal analysis, Conceptualization. **Obaidullah Ibn Raquib:** Writing – review & editing, Visualization, Formal analysis, Data curation, Conceptualization.

## Funding

This study was done with the self-funding of the principal author, Md Tamzidus Sifat, as per his thesis. No organizational or institutional funding had been accepted or received by any of the authors. No conflict of interest is present in any part of this study and the contents are solely the responsibility of the authors.

## Declaration of competing interest

The authors declare that they have no known competing financial interests or personal relationships that could have appeared to influence the work reported in this paper.

## Data Availability

Data will be made available on request.
